# The Gap Between Flare-Up Recognition and Judgment of Need for Physician Visit in Patients With Ulcerative Colitis

**DOI:** 10.4021/gr543w

**Published:** 2013-05-03

**Authors:** Makoto Tanaka, Aki Kawakami, Yasushi Iwao, Tsuneo Fukushima, Yukari Takai, Noriko Yamamoto-Mitani

**Affiliations:** a Department of Adult Nursing, Graduate School of Medicine, The University of Tokyo, 7-3-1 Hongo, Bunkyo-ku, Tokyo, 113-0033, Japan; b Center for Preventive Medicine, Keio University School of Medicine, 35 Shinano-machi, Shinjuku-ku, Tokyo, 160-8582 Japan; c Matsushima Clinic, 3-138 Ise-cho, Nishi-ku, Yokohama-shi, Kanagawa, 220-0045, Japan; d Graduate School of Health Sciences, Gunma University, 3-39-22 Showa-machi, Maebashi-shi, Gunma, 371-8514, Japan

**Keywords:** Ulcerative colitis, Inflammatory bowel disease, Self care, Patient education, Self monitoring

## Abstract

**Background:**

Although patients with ulcerative colitis (UC) recognize that their conditions are worsening, they do not always visit a doctor immediately. Our aim was to investigate how patients recognize a flare-up of UC and how significant a gap there is between symptoms recognized as a flare-up and symptoms judged to require physician visit.

**Methods:**

Questionnaires were distributed to 1,641 Crohn’s and Colitis Foundation of Japan members and returned by 426, with 260 UC patients subsequently analyzable (Crohn’s disease patients were excluded). Symptoms recognized as a flare-up of UC and symptoms judged to require physician visit were collected as free descriptions. A gap was determined if descriptions of symptoms judged to require physician visit contained expressions of prolonged symptoms, aggravation of symptoms, or critical symptoms. Furthermore, obvious delay was also determined if described symptoms contained critical symptoms.

**Results:**

Blood in stool was the most significant flare-up symptom recognized in patients with UC. A gap was observed in 134 cases (56.8%) and obvious delay was present in 70 cases (29.7%). Moreover, 52% of subjects debated whether to consult with a doctor when their conditions became subtly worse. Conversely, approximately 50% subjects also reported “If my condition subtly worsens, I want to visit a doctor immediately”.

**Conclusions:**

Although patients with UC recognized flare-ups accurately, a gap was observed in half of our subjects. Our data are important evidence that health professionals must educate patients effectively to improve patient outcomes.

## Introduction

Ulcerative colitis (UC) is an incurable disease of unknown etiology characterized by alternating periods of remission and relapse. Most patients face difficulties in their daily life due to the disease and/or treatments [1-3]. In particular, patients experience great concern regarding the possibility of unanticipated flares or feeling out of control [2, 4-6]. Starting treatment for induction of remission as soon as possible is important for patients with UC who are experiencing symptoms of relapse. Although patients recognize that their conditions are worsening by signs of relapse, they do not always visit a doctor immediately. The gap between symptoms recognized as a flare-up and symptoms judged to require physician visit might result in more serious outcomes, such as need for hospitalization. To prevent such poor outcomes, patients must be able to adequately recognize their symptoms as a flare up of UC and then visit a doctor or use additional medication as directed by a doctor to start induction treatment. In actual fact in Japan, induction treatment is usually started when a patient visits a doctor, because the concept of patient participation in treatment has not yet become widespread. Similarly, our previous study revealed that patients with Crohn’s disease (CD) hesitated to consult a doctor immediately and tried to control their disease by adjusting their daily lives, even if patients recognized that their conditions were worsening [7]. One of the possible reasons for hesitating to visit a doctor immediately is perceived severity of symptoms. Considerable numbers of patients with UC sometimes experience abdominal symptoms even in the remission phase because they have irritable bowel syndrome as a comorbidity [8-9]. Therefore, it can be difficult for patients to distinguish whether their present symptoms may be resulting from relapse of their UC. Moreover, variations in disease such as location or severity challenge health professionals to judge how to educate each UC patient about coping with a worsening in their condition. Besides, there are not enough doctors who are well-experienced in UC, because it is not a common disease in Japan. Furthermore, there is no formal guideline for educating UC patients and thus each health professional is entrusted with educating their patients according to their own professional experience. To understand how best to support UC patients, it is important to elucidate how they recognize a flare-up of their UC and how they judge whether they require physician visit. The purpose of this study was to investigate: 1). What symptoms are recognized as a flare-up of UC; and 2). How significant a gap there is between symptoms recognized as a flare-up of UC and symptoms judged to require physician visit.

## Patients and Methods

### Data collection

Data were collected by an anonymous questionnaire survey from January to June 2011. A draft questionnaire was created based on our previous studies [7] and was confirmed to have face validity by an IBD specialist. After a pilot test was conducted in 3 patients with UC, the main survey was carried out. We sent a letter of explanation of the survey and the questionnaire to members of the Crohn’s and Colitis Foundation of Japan (CCFJ), which is a nonprofit, volunteer-run organization dedicated to finding cures for CD and UC [10]. The return of a questionnaire implied consent for participation in the survey. This study was conducted with the approval of the ethics committee of The University of Tokyo.

The questionnaire explored symptoms recognized as indicating a flare-up of UC and symptoms judged to require physician visit, and included questions on cognitions about consulting with a doctor when their conditions are worsening, disease characteristics, and demographic characteristics. We investigated as follows. 1). Symptoms recognized as a flare-up of UC and symptoms judged to require physician visit were elicited by open-ended questions. 2). Cognitions about consulting with a doctor when their symptoms are worsening were elicited by five questions with answers obtained by ordinal scale responses (“1 = completely disagree” to “5 = completely agree”). 3). Disease characteristics elicited were duration of morbidity, current treatments, and treatment experience. 4). Demographic characteristics elicited were age, sex, occupational status, and marital status.

### Data analysis

We used the content analysis method [11] to summarize the contents of the free description about symptoms. The gap between symptoms recognized as a flare-up and symptoms judged to require physician visit was analyzed by comparing these described symptoms, and the presence of a gap was determined if descriptions of symptoms judged to require physician visit contained expressions of prolonged symptoms, aggravation of symptoms, or critical symptoms. Furthermore, obvious delay in visiting a doctor was also defined if described symptoms contained one of the following expressions of severe symptoms: bloody diarrhea more than 6 times per day, blood in stool accompanying fever, bloody diarrhea for more than 1 week, and problems interfering with life such as nocturnal bowel movements, being unable to take food, or weight loss. Initially, the first author (M.T.) conducted the coding carefully for all of our data. Subsequently, two other coders determined the code for each description independently for 25.4% of samples selected conveniently (picked up in serial order) from all the data. The concordance rates for determinations of the codes by the two independent coders were 81.6-84.1%. Finally, the confirmed codes were counted and handled as descriptive data. Statistical analyses were performed using IBM SPSS statistics version 19 for Windows.

## Results

### Subjects

The questionnaires were distributed to 1,641 CCFJ members and 426 (recovery rate was 26%) patients returned the questionnaire. One hundred forty CD patients and four patients of unknown diagnosis were excluded (this study was conducted as a part of research project of self-management in patients with IBD). Furthermore, 22 UC patients who had undergone surgery were also excluded. Therefore, 260 UC patients were analyzable and their characteristics are reported in Table 1. There were 143 married persons (55.2%) and 117 unmarried persons (44.8%). There were 148 subjects (56.9%) with a job and 13 students (5.0%).

**Table 1:** Self-Reported Clinical Characteristics and Demographic Parameters

Variable	Mean ± SD		Case (%) (n=260)
Age (y)	47.2 ± 16.1		
Disease duration (y)	12.8 ± 7.9		
Gender (male)			130 (50.0)
Current treatment			
Oral aminosalicylates			217 (83.8)
Topical aminosalicylates			48 (18.5)
Oral corticosteroid			32 (12.3)
Topical corticosteroid			35 (13.5)
Immunomodulatory drug (6MP, AZA)			34 (13.1)
Immunomodulatory drug (Tacrolimus)			1 (0.4)
Leukocyte apheresis			5 (1.9)
Biologics			7 (2.7)
Supplemental elemental diet			11 (4.2)
Treatment Experience			
Oral aminosalicylates			240 (92.7)
Topical aminosalicylates			160 (61.5)
Oral corticosteroid			177 (68.1)
Topical corticosteroid			175 (67.3)
Immunomodulatory drug (6MP, AZA)			69 (26.5)
Immunomodulatory drug (Tacrolimus)			13 (5.0)
Leukocyte apheresis			86 (33.1)
Biologics			9 (3.5)
Supplemental elemental diet			60 (23.1)

### Symptoms recognized as flare up of UC and symptoms judged to require physician visit

Table 2 shows the content analysis of the free description about symptoms recognized as a flare-up of UC. Two hundred forty-seven UC patients described the symptoms recognized as a flare-up. Those listed in the top half of Table 2 were reported by at least 10 subjects. Blood in stool was the most described symptom, followed by frequent bowel movements. Aside from symptoms listed in the top half of Table 2, there were various symptoms from subtle changes, such as smell of stool (8 cases), bowel sounds (6 cases), or loss of appetite (4 cases), to crucial symptoms, such as weight loss (3 cases), nocturnal bowel movements (3 cases), or erythema nodosum (2 cases).

**Table 2:** Symptoms Recognized as a Flare-Up of UC and Symptoms Judged to Require Physician Consultation

Symptoms recognized as a flare-up of UC		(n = 247)
	n	%
Blood in stool	145	58.7
Frequent bowel movements	87	35.2
Stool mucus	83	33.6
Abdominal pain	76	30.8
Diarrhea	58	23.5
Loose stool	29	11.7
Fatigue	25	10.1
Urgency	23	9.3
Fever	20	8.1
Flatulence	16	6.5
Tenesmus	12	4.9
Abdominal discomfort	10	4.0

Symptoms judged to require physician consultation		(n = 237)
	n	%
Blood in stool	156	65.8
(worsening of bloody stool)	(38)	
Frequent bowel movements	84	35.4
(more than 6 times per day)	(18)	
Abdominal pain	56	23.6
Diarrhea	35	14.8
Stool mucus	21	8.9
Fever	20	8.4
Fatigue	10	4.2

Concerning symptoms judged to require physician visit, 237 UC patients described the symptoms listed in the bottom half of Table 2. Similarly, the most-described symptom was blood in stool, which was reported by two-thirds of subjects (156 cases), followed by frequent bowel movements (84 cases). Among the 156 cases, 38 cases expressed worsening of bloody stools.

**Figure 1:**
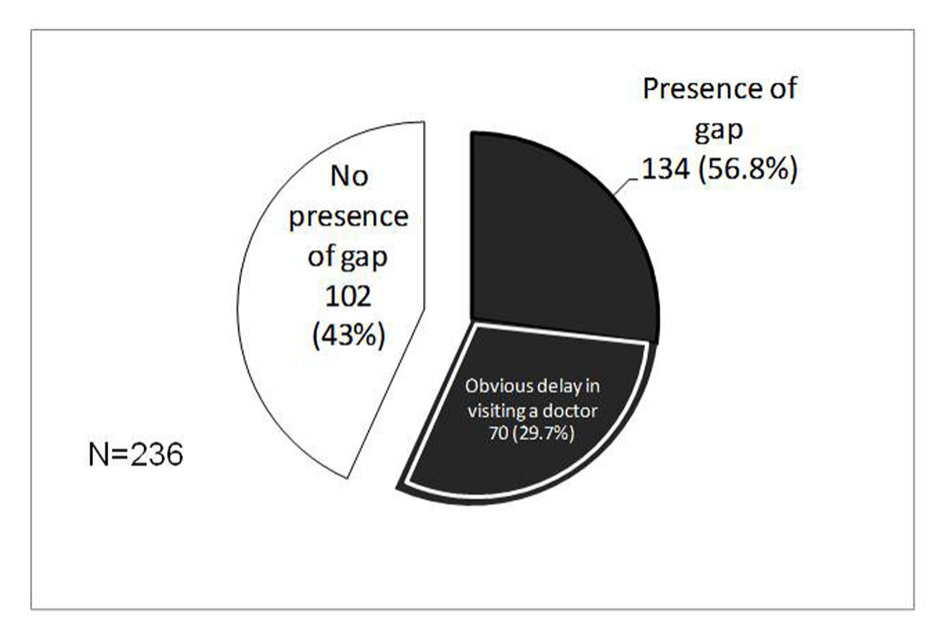
Presence of gap between symptoms recognized as a flare-up of UC and symptoms judged to require physician visit.

Figure 1 shows the analysis for presence of a gap. There were 236 pairs of descriptions concerning symptoms recognized as a flare-up and judged to require physician visit. Among them, 134 cases (56.8%) were coded as having the presence of a gap. Among them, there were also some valid gaps, such as early recognition of a flare-up. Conversely, 70 cases (29.7%) were coded as having the presence of obvious delay in visiting a doctor. Beyond those cases, many cases were not judged to have the presence of an obvious delay in visit, because of no clear notification about frequency of bowel movement or duration of prolonged critical symptoms.

### Cognition about consulting a doctor when their symptoms are worsening

**Figure 2:**
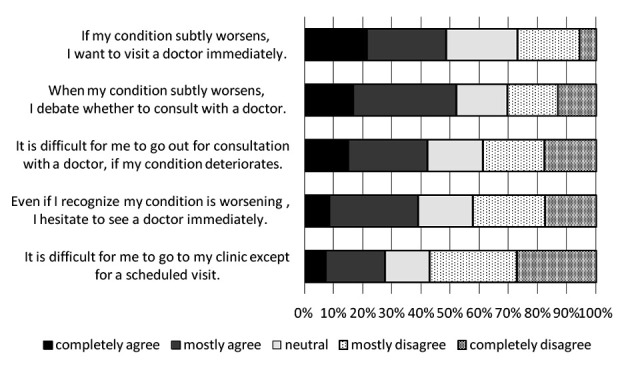
Recognition of the need to consult a doctor when symptoms are worsening.

Figure 2 shows the descriptive results of cognitions about consulting a doctor when their symptoms are worsening. The total percentage of subjects who answered “when my condition subtly worsens, I debate whether to consult with a doctor” with “completely agree” or “agree” was over 52%. Conversely, the total percentage of subjects who reported “completely agree” or “agree” concerning the cognition of “If my condition subtly worsens, I want to visit a doctor immediately” was also approximately 50%.

## Discussion

We elucidated that blood in stool was the most significant symptom recognized as a flare-up in patients with UC, and that there was a gap between symptoms recognized as a flare-up of UC and symptoms judged to require physician visit in more than half of our subjects. Moreover, approximately 50% of subjects debated whether to consult with a doctor. Blood in stool is one the most UC-specific symptoms [12], which is contained in almost all clinical disease activity indices of UC [13]. Therefore, our findings suggest that in spite of patients’ valid recognitions of their flare-ups, there could have been significant obvious delays in starting induction therapy. Our data are important evidence that our health professionals must educate, involve, and empower patients so that their UC can be provided with induction treatment earlier when they experience symptom of relapse. Some studies in the US and Europe have demonstrated improved patient outcomes [14-17] or health care service use [14-15] by giving personalized self-management plans, providing self-monitoring tools, and clearly indicating some circumstances for which patients should make a clinic appointment. However, there has been no study focusing on patient education or patient coping with flare-ups of UC in Asia, in spite of increasing numbers of patients with UC [18-20].

We think that the gap does not necessarily mean a delay which creates a problem. Early recognition of a flare-up by subtle signs usually results in a valid gap, while the higher cognition about preference for immediately consulting with a doctor occasionally leads to some inappropriate immediate visits. Other than blood in stool, abdominal pain and frequent bowel movements are among the common symptoms experienced for various other reasons such as IBS or virus infection and therefore patients may have difficulty distinguishing a relapse of UC from these symptoms alone. Furthermore, abdominal pain or frequent bowel movements may make UC patients hesitant to go out. Also, some patients may add extra medication when they recognize a flare-up by early signs. On an empirical basis, some Japanese experts in IBD provide their patients with a personalized self-management plan, especially topical therapies that are easy to entrust to patients. However, the present study could not take into account directions about how to cope with flare-ups from healthcare providers. These might influence patient perceptions of circumstances where they should consult with a doctor. In the future, we must investigate the actual state of patient education about how to cope with flare-ups of UC.

There were approximately 30% of patients who still did not visit a doctor until they experienced severe symptoms. This might be because more than half of subjects debated whether to consult a doctor and more than quarter of subjects experienced difficulty in going to the clinic except for a scheduled visit. If we could include further information about what patients do when they have recognized a flare-up, our findings might become more valuable and our definition of obvious delay might not fit in mild or moderate relapse cases. However, to diagnose a problem concerning delay by using descriptions of symptoms is difficult, due to the absence of clinical background data or disease history information. Although the presence of a gap might over-diagnose the problem and obvious delay might also under-diagnose the problem, both outcomes have contributed to identifying and highlighting the problem.

The present study had several limitations. First, the study was conducted in the membership of the CCFJ, which is a nonprofit, volunteer-run organization dedicated to finding cures for CD and UC [10]. We should note that the results of the present study were based on a sample containing patients with a comparatively high concern about their diseases. Because all of our subjects might not be followed by a doctor who is an IBD expert, if the survey was conducted in other settings, such as an IBD center, the results would be different. Hence, this sample is not sufficiently representative for our results to be generalized. Next, all data were collected by self-report, and examination of clinical data was insufficient. Exploration of factors related to the gap could not be exhaustive. Furthermore, symptoms recognized as a flare-up and symptoms judged to require physician visit were collected by free description, and therefore patients may have omitted or over-exaggerated some symptoms. Consequently, both under-estimates and over-estimates of some problems might have occurred. However, we found that a considerable number of patients with UC possess a problem about the timing of physician visit. We should provide effective patient education which can improve patient outcomes. To do that, further studies that develop a valuable education program for coping with worsening conditions are necessary. After that, we should examine the efficacy of the program for the sake of understanding effective support for UC patients. Although these are the tasks ahead, this study is valuable given that there was no previous study in patients with UC about their perception of circumstances when they should consult a doctor.

In conclusion, the present study revealed that although patients with UC recognized flare-ups accurately, there was a gap between symptoms recognized as a flare-up of UC and symptoms judged to require physician visit in more than half of our subjects. The presence of obvious delays in visiting a doctor was observed in approximately thirty percent of our subjects. We should provide effective education for these patients. These findings will help to understand UC patients and to develop a patient education program to improve the timing of starting induction treatment.

## References

[1] Mitchell A, Guyatt G, Singer J, Irvine EJ, Goodacre R, Tompkins C, Williams N (1988). Quality of life in patients with inflammatory bowel disease. J Clin Gastroenterol.

[2] Drossman DA, Patrick DL, Mitchell CM, Zagami EA, Appelbaum MI (1989). Health-related quality of life in inflammatory bowel disease. Functional status and patient worries and concerns. Dig Dis Sci.

[3] Joachim G, Milne B (1987). Inflammatory bowel disease: effects on lifestyle. J Adv Nurs.

[4] Levenstein S, Li Z, Almer S, Barbosa A, Marquis P, Moser G, Sperber A (2001). Cross-cultural variation in disease-related concerns among patients with inflammatory bowel disease. Am J Gastroenterol.

[5] Mussell M, Bocker U, Nagel N, Singer MV (2004). Predictors of disease-related concerns and other aspects of health-related quality of life in outpatients with inflammatory bowel disease. Eur J Gastroenterol Hepatol.

[6] Irvine EJ (2004). Review article: patients' fears and unmet needs in inflammatory bowel disease. Aliment Pharmacol Ther.

[7] Tanaka M, Iwao Y, Okamoto S, Ogata H, Hibi T, Kazuma K (2009). Coping strategy when patients with quiescent Crohn's disease recognize that their conditions are worsening. J Gastroenterol.

[8] Simren M, Axelsson J, Gillberg R, Abrahamsson H, Svedlund J, Bjornsson ES (2002). Quality of life in inflammatory bowel disease in remission: the impact of IBS-like symptoms and associated psychological factors. Am J Gastroenterol.

[9] Minderhoud IM, Oldenburg B, Wismeijer JA, van Berge Henegouwen GP, Smout AJ (2004). IBS-like symptoms in patients with inflammatory bowel disease in remission; relationships with quality of life and coping behavior. Dig Dis Sci.

[10] http://www.ccfj.jp/index.htm.

[11] Krippendorff K (1989). Content analysis: An introduction to its methodology (Japanese Translation).

[12] Joyce JC, Waljee AK, Khan T, Wren PA, Dave M, Zimmermann EM, Wang S (2008). Identification of symptom domains in ulcerative colitis that occur frequently during flares and are responsive to changes in disease activity. Health Qual Life Outcomes.

[13] D'Haens G, Sandborn WJ, Feagan BG, Geboes K, Hanauer SB, Irvine EJ, Lemann M (2007). A review of activity indices and efficacy end points for clinical trials of medical therapy in adults with ulcerative colitis. Gastroenterology.

[14] Robinson A, Thompson DG, Wilkin D, Roberts C (2001). Guided self-management and patient-directed follow-up of ulcerative colitis: a randomised trial. Lancet.

[15] Kennedy AP, Nelson E, Reeves D, Richardson G, Roberts C, Robinson A, Rogers AE (2004). A randomised controlled trial to assess the effectiveness and cost of a patient orientated self management approach to chronic inflammatory bowel disease. Gut.

[16] Elkjaer M, Shuhaibar M, Burisch J, Bailey Y, Scherfig H, Laugesen B, Avnstrom S (2010). E-health empowers patients with ulcerative colitis: a randomised controlled trial of the web-guided 'Constant-care' approach. Gut.

[17] Cross RK, Cheevers N, Finkelstein J (2009). Home telemanagement for patients with ulcerative colitis (UC HAT). Dig Dis Sci.

[18] Asakura K, Nishiwaki Y, Inoue N, Hibi T, Watanabe M, Takebayashi T (2009). Prevalence of ulcerative colitis and Crohn's disease in Japan. J Gastroenterol.

[19] Molodecky NA, Soon IS, Rabi DM, Ghali WA, Ferris M, Chernoff G, Benchimol EI (2012). Increasing incidence and prevalence of the inflammatory bowel diseases with time, based on systematic review. Gastroenterology.

[20] Ooi CJ, Fock KM, Makharia GK, Goh KL, Ling KL, Hilmi I, Lim WC (2010). The Asia-Pacific consensus on ulcerative colitis. J Gastroenterol Hepatol.

